# Multiphoton imaging of myogenic differentiation in gelatin-based hydrogels as tissue engineering scaffolds

**DOI:** 10.1186/s40824-016-0050-x

**Published:** 2016-01-18

**Authors:** Min Jeong Kim, Yong Cheol Shin, Jong Ho Lee, Seung Won Jun, Chang-Seok Kim, Yunki Lee, Jong-Chul Park, Soo-Hong Lee, Ki Dong Park, Dong-Wook Han

**Affiliations:** Department of Cogno-Mechatronics Engineering, College of Nanoscience & Nanotechnology, Pusan National University, Busan, 46241 Korea; Department of Molecular Science and Technology, Ajou University, Suwon, 16499 Korea; Department of Medical Engineering, Cellbiocontrol Laboratory, Yonsei University College of Medicine, Seoul, 03722 Korea; Department of Biomedical Science, CHA University, Gyeonggi-do, 11160 Korea

**Keywords:** Hydrogel, 3D scaffolds, Multiphoton microscopy, C2C12 myoblast, Myogenic differentiation

## Abstract

**Background:**

Hydrogels can serve as three-dimensional (3D) scaffolds for cell culture and be readily injected into the body. Recent advances in the image technology for 3D scaffolds like hydrogels have attracted considerable attention to overcome the drawbacks of ordinary imaging technologies such as optical and fluorescence microscopy. Multiphoton microscopy (MPM) is an effective method based on the excitation of two-photons. In the present study, C2C12 myoblasts differentiated in 3D gelatin hydroxyphenylpropionic acid (GHPA) hydrogels were imaged by using a custom-built multiphoton excitation fluorescence microscopy to compare the difference in the imaging capacity between conventional microscopy and MPM.

**Results:**

The physicochemical properties of GHPA hydrogels were characterized by using scanning electron microscopy and Fourier-transform infrared spectroscopy. In addition, the cell viability and proliferation of C2C12 myoblasts cultured in the GHPA hydrogels were analyzed by using Live/Dead Cell and CCK-8 assays, respectively. It was found that C2C12 cells were well grown and normally proliferated in the hydrogels. Furthermore, the hydrogels were shown to be suitable to facilitate the myogenic differentiation of C2C12 cells incubated in differentiation media, which had been corroborated by MPM. It was very hard to get clear images from a fluorescence microscope.

**Conclusions:**

Our findings suggest that the gelatin-based hydrogels can be beneficially utilized as 3D scaffolds for skeletal muscle engineering and that MPM can be effectively applied to imaging technology for tissue regeneration.

## Background

Recently, the development of medical image technology for biomedical applications has attracted considerable interest [1]. Conventional image technologies, including optical microscopy and confocal microscopy have contributed significantly to the development of biology and biomedicine [2–5]. On the other hand, the conventional image technologies have several disadvantages, such as light attenuation in tissue and limited light transparency. Multiphoton microscopy (MPM) is an effective method to overcome the limitations of conventional technologies [6].

MPM is a powerful tool that is based on the excitation of two-photons [7]. Confocal microscope a useful tool to obtain three-dimensional (3D) image, is technically similar to MPM. However, it has limitation to observe thick specimens because the scattering of excitation and emission photons. Also, the absorption of the excitation light induced the photo-bleaching. On the other hand, MPM can measure the deeper layers of tissue due to a decrease in light attenuation and minimize the photo-damage site [8–11]. In addition, this technology improves the signal to background ratio as well as the sensitivity and spatial resolution. Therefore, MPM has attracted considerable attention as a superior image technology. This non-linear optical phenomenon allows one to obtain a three dimensional image of a cell and tissue [12–16]. The custom-built MPM used in this study consists of a femtosecond (fs) titanium:sapphire laser, polarizer, x-y scanner, beam expander, dichoic mirror, photomultiplier tube (PMT), discriminator, and etc. The nominal value of the laser pulse-width was approximately 120 fs and the in situ average power was between 5 to 50 mW.

On the other hand, the development of a biomimetic artificial scaffold for tissue engineering applications using a range of biomaterials has attracted interest. Among the many biomaterials, hydrogels are used widely to culture cells in 3D [17–19]. A hydrogel can be injected into the body to simultaneously synthesize a scaffold [20–22]. In particular, the hydrogel used in our study can be easily controlled the mechanical properties including stiffness by adjusting the concentration of hydrogen peroxide (H_2_O_2_) and horseradish peroxidase (HRP) [23, 24]. When the cells are cultured in hydrogel-based scaffolds, they are grown inside the hydrogel in 3D. Especially, gelatin-based hydrogels have excellent biocompatibility and non-immunogenicity, and also possess RGD sequences, which support the cell adhesion, proliferation and differentiation [25–30]. Although there are many systems for observing the cellular behaviors, there are limitations and restrictions to the observation of cellular behaviors using 2D imaging systems. Therefore, to address this limitation, MPM was used to observe cells growing inside a gelatin-based hydrogel in 3D.

In the present study, gelatin-based hydrogels were prepared and their physicochemical properties were characterized. The chemical composition of the hydrogels was characterized by Fourier-transform infrared (FT-IR) spectroscopy. The morphology of the hydrogels was observed by scanning electron microscopy (SEM). In addition, C2C12 myoblasts were cultured in the hydrogels and incubated in growth media (GM) and differentiation media (DM). Immunofluorescence analysis was conducted for the myosin heavy chain (MHC) of C2C12 myoblasts to examine the myogenic differentiation of myoblasts. The stained cells were imaged by custom-built multiphoton excitation fluorescence microscopy.

## Methods

### Materials

Gelatin hydroxyphenylpropionic acid (GHPA) conjugates were synthesized using the procedure described elsewhere (Fig. 2) [19, 21]. Briefly, gelatin was dissolved in 40 °C deionized water for 1 hour. Hydroxyphenylpropionic acid (HPA) was dissolved in water and dimethylformamide (DMF) then mixed with 1-ethyl-3-(3-dimethylaminopropyl)-carbodiimide (EDC) and *N*-hydroxysuccinimide (NHS). After then, HPA solution was added to gelatin solution and stirred at 40 °C for 1 day. The resulting solution was dialyzed deionized water for 3 days and subjected to the dialysis in NaCl, water/ethanol and distilled water, subsequently. The purified solution was freeze-dried to obtain the GHPA conjugates. Peroxidase from horseradish (type VI, Sigma-Aldrich Co., St. Louis, MO) and H_2_O_2_ (30 wt. % in H_2_O, Junsei Chemical Co., Tokyo, Japan) were used for cross-linking the GHPA hydrogel.

### Cell culture

C2C12 myoblasts (derived from thigh muscle of C3H mice) were obtained from the American Type Culture Collection (Rockville, MD) and maintained routinely in GM, Dulbecco’s modified Eagle’s Medium (DMEM, Welgene, Daegu, Korea) complemented with 10 % fetal bovine serum (Welgene) and a 1 % antibiotic-antimycotic solution (including 10,000 units penicillin, 10 mg streptomycin and 25 μg amphotericin B per mL, Sigma-Aldrich Co.). DM for myogenic differentiation is a low-serum media [DMEM containing 2 % of horse serum (Welgene) and 1 % antibiotic-antimycotic solution]. Live and dead cell assay was conducted by using the Live/Dead Viability/Cytotoxicity Kit (Molecular Probes, Eugene, OR). A cell counting kit-8 (CCK-8, Dojindo, Kumamoto, Japan) was used to evaluate the proliferation of C2C12 myoblasts in GHPA hydrogels.

### Physicochemical characterizations of GHPA hydrogels

The morphology of the GHPA hydrogels was examined by SEM (S-800, Hitachi, Tokyo, Japan). The GHPA hydrogels were prepared in a cell culture slide (SPL life science, Gyeonggi-do, Korea) by mixing 100 μL of 3 % GHPA containing HRP (0.005 mg/mL) and H_2_O_2_ (0.01 wt. %). The cross-linked hydrogels were freeze-dried over a 2 day period. The dehydrated hydrogels were sputter coated with an ultrathin layer of gold/platinum by ion sputtering (E1010, Hitach, Tokyo, Japan) prior to the SEM observations.

Compositional analysis of the GHPA hydrogel was performed by FT-IR spectroscopy (Nicolet 560, Nicolet Co., Madison, WI). The GHPA hydrogel was prepared on a slide glass covered with cover glass.

### Live and dead cell assay of C2C12 cells in GHPA hydrogels

A live and dead cell assay was implemented to measure the viability of the C2C12 myoblasts in the GHPA hydrogel. The C2C12 myoblasts were seeded in GHPA hydrogel at a density of 1 × 10^5^ cells/mL and incubated in GM. The GM was changed every 2-3 days. After 1 and 7 days of culture, the live and dead cell assay solution (2 μM calcein AM and 4 μM ethidium homodimer-1) was added to each cell-seeded hydrogel and incubated for 30 min at 37 °C. The cells were then observed using Olympus IX81 inverted fluorescence microscope (Olympus Corp, Osaka, Japan).

### Proliferation of C2C12 cells in GHPA hydrogels

To evaluate the proliferation of the C2C12 myoblasts in GHPA hydrogel, the C2C12 myoblasts were seeded in GHPA hydrogels at a density of 1 × 10^5^ cells/mL and each cell-seeded hydrogel was incubated with a CCK-8 solution in the last 4 hours of the culture periods (1, 3, 5, and 7 days) at 37 °C in the dark. The absorbance was measured at 450 nm using an ELISA reader (SpectraMax® 340, Molecular Device Co, Sunnyvale, CA).

### Immunostaining of C2C12 cells in GHPA hydrogels

To examine the myogenic differentiation, C2C12 myoblasts were seeded in GHPA hydrogels at a density of 1 × 10^5^ cells/mL and incubated in GM for 4 days. The cells were then incubated for an additional 3 days in GM and DM, respectively. The cells were fixed with a 3.7 % formaldehyde solution (Sigma-Aldrich Co.) for 10 min and permeabilized with 0.1 % Triton X-100 (Sigma-Aldrich Co.) for 5 min. Subsequently, the cells were blocked with a 2 % bovine serum albumin (BSA, GenDEPOT, Barker, TX) solution in Dulbecco’s phosphate-buffered saline (DPBS, Gibco BRL, Rockville, MD) for 1 hour and incubated with Alexa Fluor 488-conjugated anti-MHC monoclonal antibody (1:200 in 1 % BSA solution in DPBS, eBioscienceInc., San Diego, CA) overnight at 4 °C. Subsequently, the cells were incubated with TRITC-labeled phalloidin (200 units/mL in methanol, 1:40 in 1 % BSA solution in DPBS, Molecular Probes, Eugene, OR) for 1 hour at room temperature. The stained cells were imaged using an Olympus IX81 inverted fluorescence microscope and a custom-built MPM. The fluorescence images were analyzed using ImageJ software (National Institutes of Health, Bethesda, MD).

### Custom-built multiphoton microscopy

**Fig. 1:**
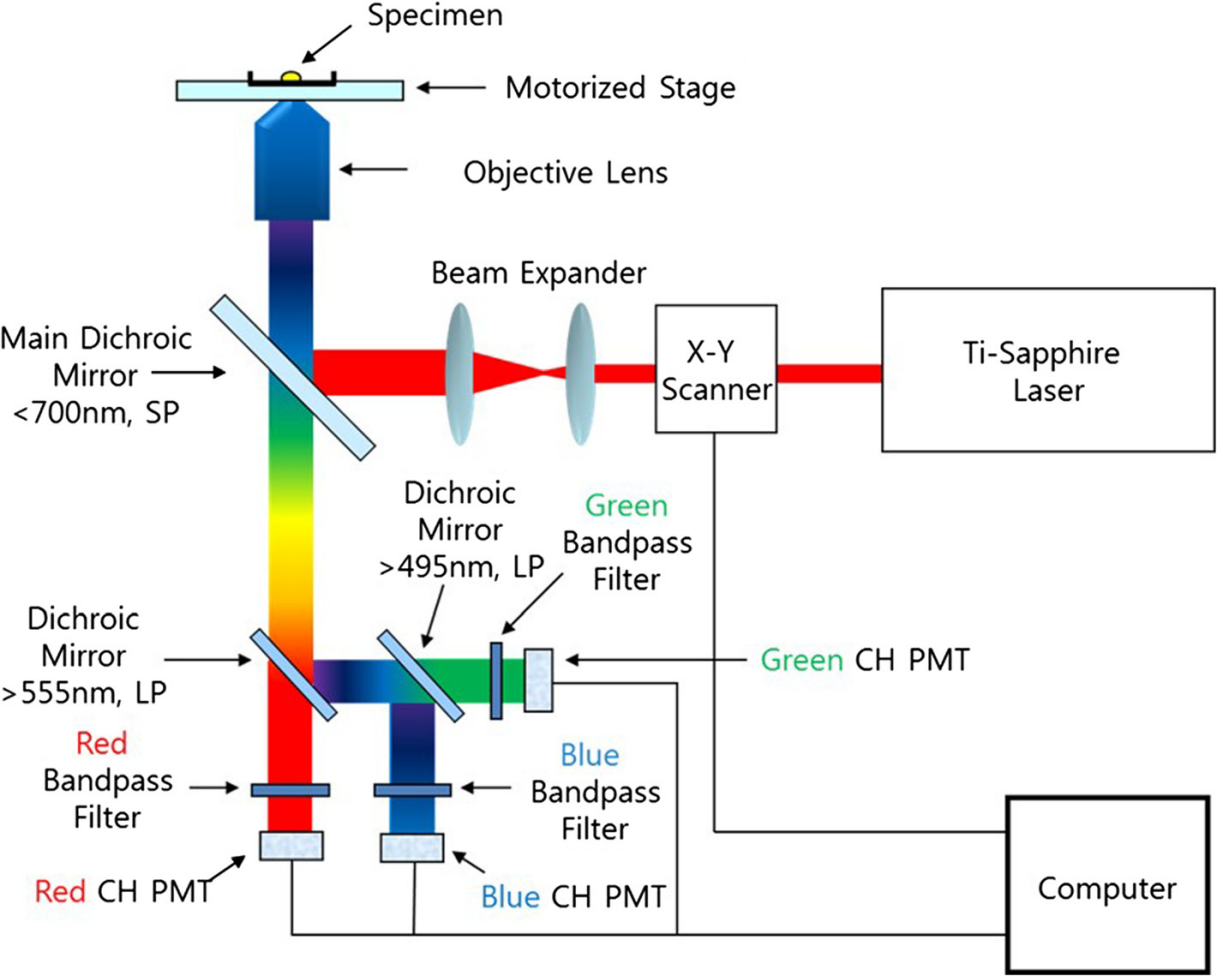
Schematic diagram of the MPM imaging system used in this study. The custom-built MPM is composed of a femtosecond (fs) titanium:sapphire laser, polarizer, x-y scanner, beam expander, dichoic mirror, photomultiplier tube (PMT), discriminator, and etc

A custom-built MPM was used to obtain morphological and functional information of the C2C12 myoblasts in the GHPA hydrogels. Figure 1 shows a schematic diagram of the custom-built MPM. In this custom-built MPM, laser sources of a Ti-sapphire tunable femtosecond pulse laser (680-1080 nm, 140 fs, 80 MHz) were used. Raster scanning was performed using a galvo x-y mirror to obtain a 2D image and z-stacking was performed using a motorized stage to acquire 3D images. The laser beam was expanded by a beam expander and reflected onto the back aperture of the objective lens by a short pass main dichroic mirror (T700DCSPXRUV, Chroma Technology). The beam was focused onto the C2C12 myoblasts in the GHPA hydrogels by the objective lens (UPLFLN 10XP, Olympus). A two-photon induced fluorescence signal was produced by a focused beam. The fluorescence signal from the C2C12 myoblasts in GHPA hydrogels was recollected in the backward direction by focusing the objective lens and further guided by long pass dichroic mirrors set for three channels: red, green and blue. In each color case, the fluorescence signals were isolated by band pass filters and detected by photomultiplier tubes (H10682-210, Hamamatsu Photonics).

### Statistical analysis

All variables were tested in three independent cultures for each experiment, which was repeated twice (*n* = 6). All the quantitative data is expressed as the mean ± standard deviation (SD). Statistical comparisons were carried out by a one-way analysis of variance (ANOVA), followed by a Bonferroni test for multiple comparisons. A value of *p* < 0.05 was considered statistically significant.

## Results and discussion

### Characterizations of GHPA hydrogels

**Fig. 2:**
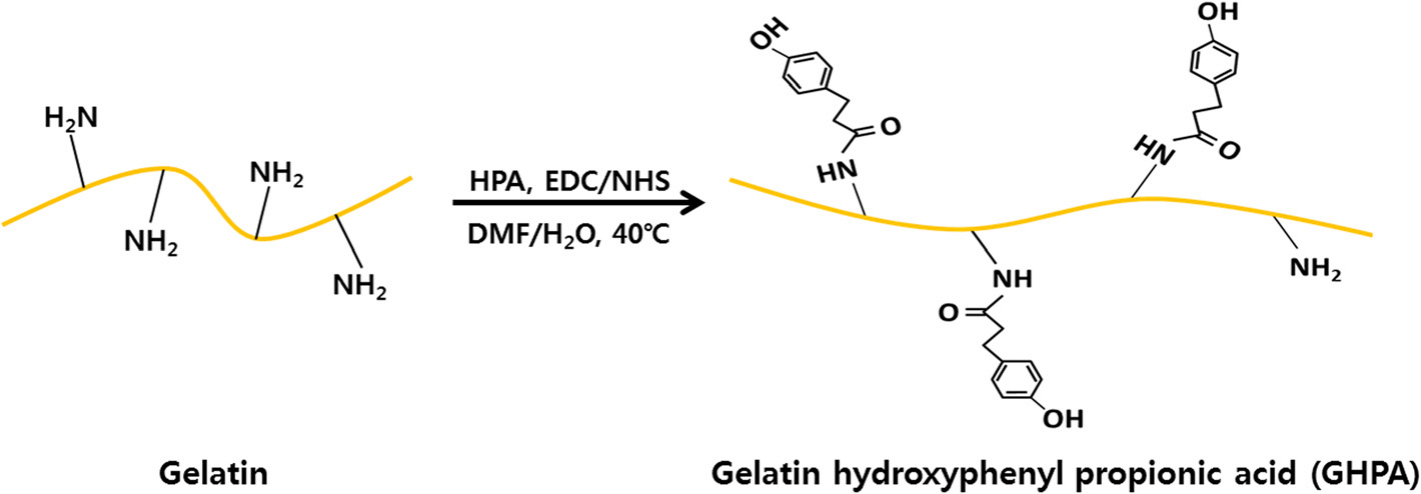
Schematic diagram of GHPA hydrogel synthesis. Synthesis and chemical structure of GHPA hydrogel

**Fig. 3:**
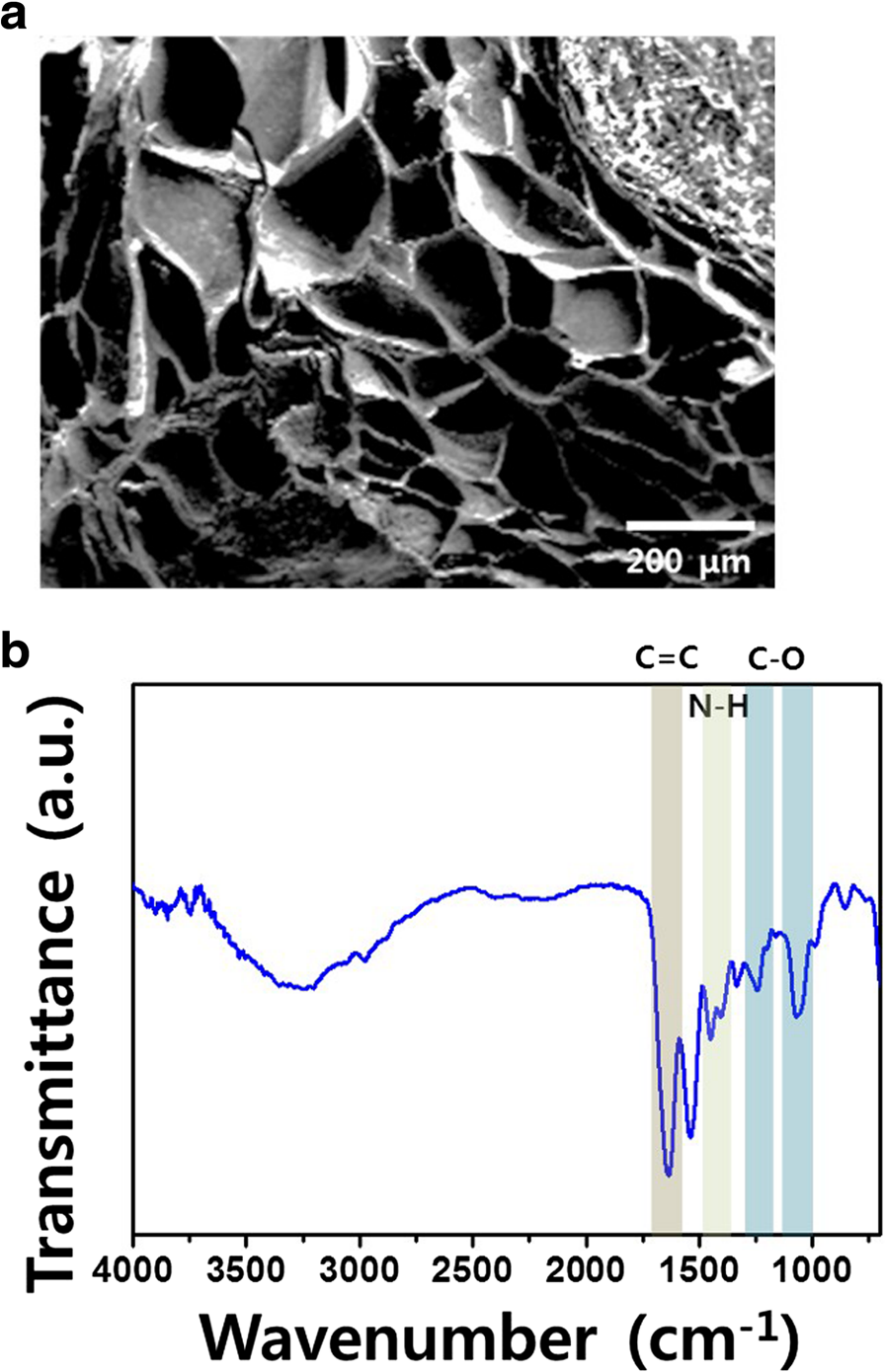
Cross-sectional morphological images and FT-IR spectrum of GHPA hydrogel. Representative SEM image of GHPA hydrogels (**a**). Representative FT-IR spectrum of GHPA (**b**). All images shown in this figure are representative of six independent experiments with similar results

The GHPA hydrogels were cross-linked enzymatically by HRP and H_2_O_2_. The cross-linking forms the network mesh structure in the hydrogel. The network mesh structure of the hydrogels allows a high water uptake [23, 31]. The high water uptake ability and appropriate pore size of hydrogels can facilitate the rapid diffusion of nutrients in the media and cell metabolites. The pore size of the GHPA hydrogel can be controlled by adjusting the concentration of HRP and H_2_O_2_ [23]. Figure 2 (a) shows a cross-section image of the GHPA hydrogel. The cross-sectional image of the GHPA hydrogel showed that the GHPA hydrogel had a network mesh structure and a mean pore size in the range between 50 and 100 μm. A network mesh structure of the hydrogels allows them to have excellent water permeability/retention [19, 32, 33]. Figure 3 (b) shows a FT-IR spectrum of the GHPA hydrogel. A strong band was observed at 1634 cm^-1^, which represents C = C aromatic bonding [34]. The characteristic band was observed at 1540 cm^-1^, which was attributed to the N-H bending and C-N stretching [35]. The specific bands were observed at 1390, 1240 and 1067 cm^-1^, which were assigned to the C-O bonding, epoxy stretching and alkoxy stretching, respectively [34–37].

### Live and dead cell assay and proliferation of C2C12 cells in GHPA hydrogels

**Fig. 4:**
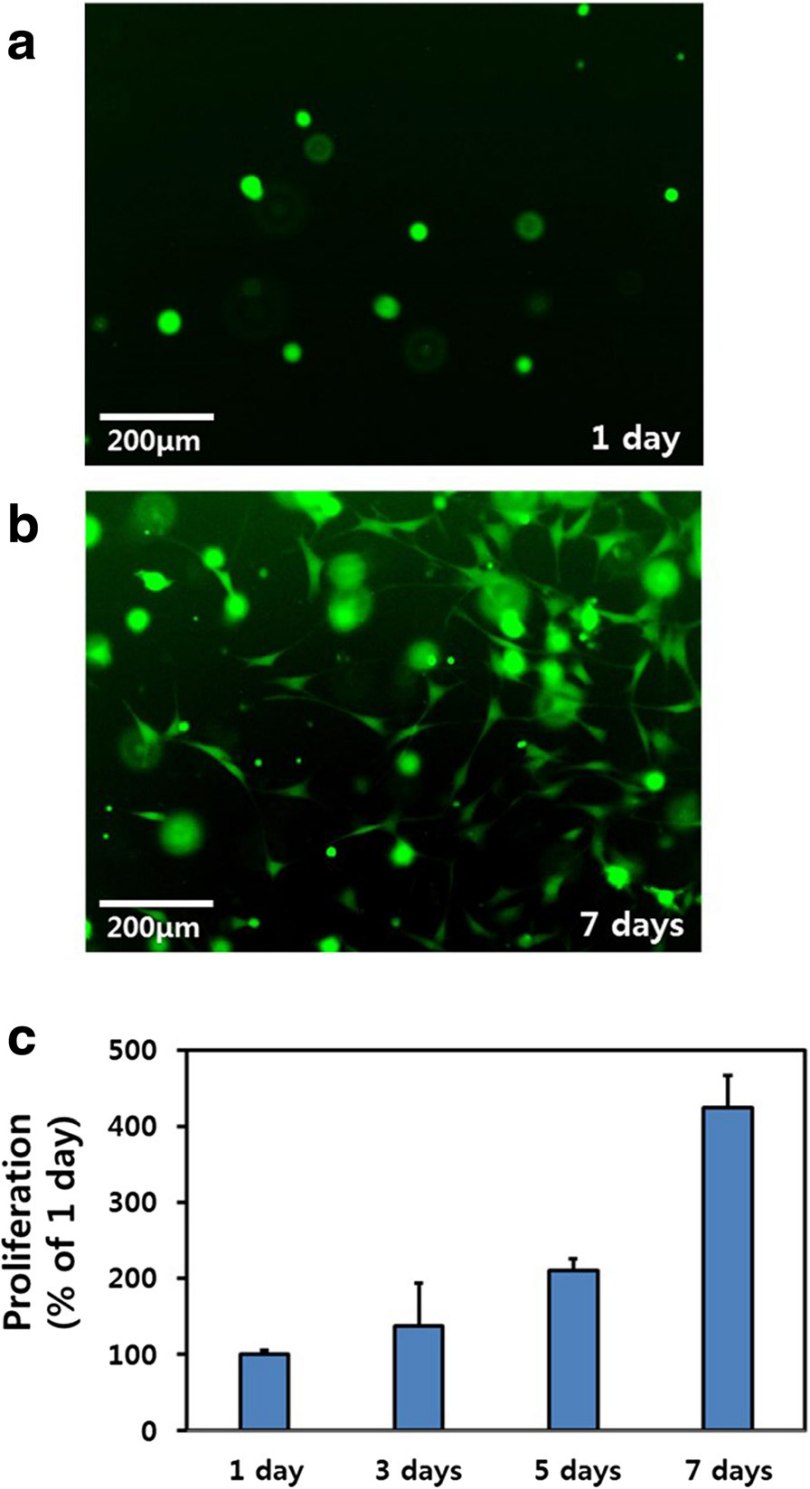
Live and dead cell assay analysis and proliferation graph of C2C12 cells in hydrogels. Live and dead cell assay results of C2C12 myoblasts cultured in GHPA hydrogels on 1 day (**a**) and 7 days (**b**)**.** After culture and the proliferation of C2C12 myoblasts cultured in GHPA hydrogels (**c**). All images shown in this figure are representative of six independent experiments with similar results

Figures 4 (a) and (b) show the live and dead cell assay results of the C2C12 myoblasts in GHPA hydrogels at 1 and 7 days after culture, respectively. The green fluorescence from the live cells was detected, indicating that the cells had spread well in the GHPA hydrogel. Regarding the cell viability, the cells in the hydrogel were viable (stained green), even after 7 days of culture, and no dead cells were detected (stained red). The proliferation of C2C12 myoblasts in the GHPA hydrogels was evaluated using a CCK-8 assay, as shown in Fig. 4 (c). The proliferation of C2C12 myoblasts in the GHPA hydrogels was increased gradually over a 7 day period. These results suggest that the GHPA hydrogels can provide a suitable 3D microenvironment for C2C12 myoblast growth.

### Immunofluorescence staining analysis of C2C12 cells in GHPA hydrogels

**Fig. 5:**
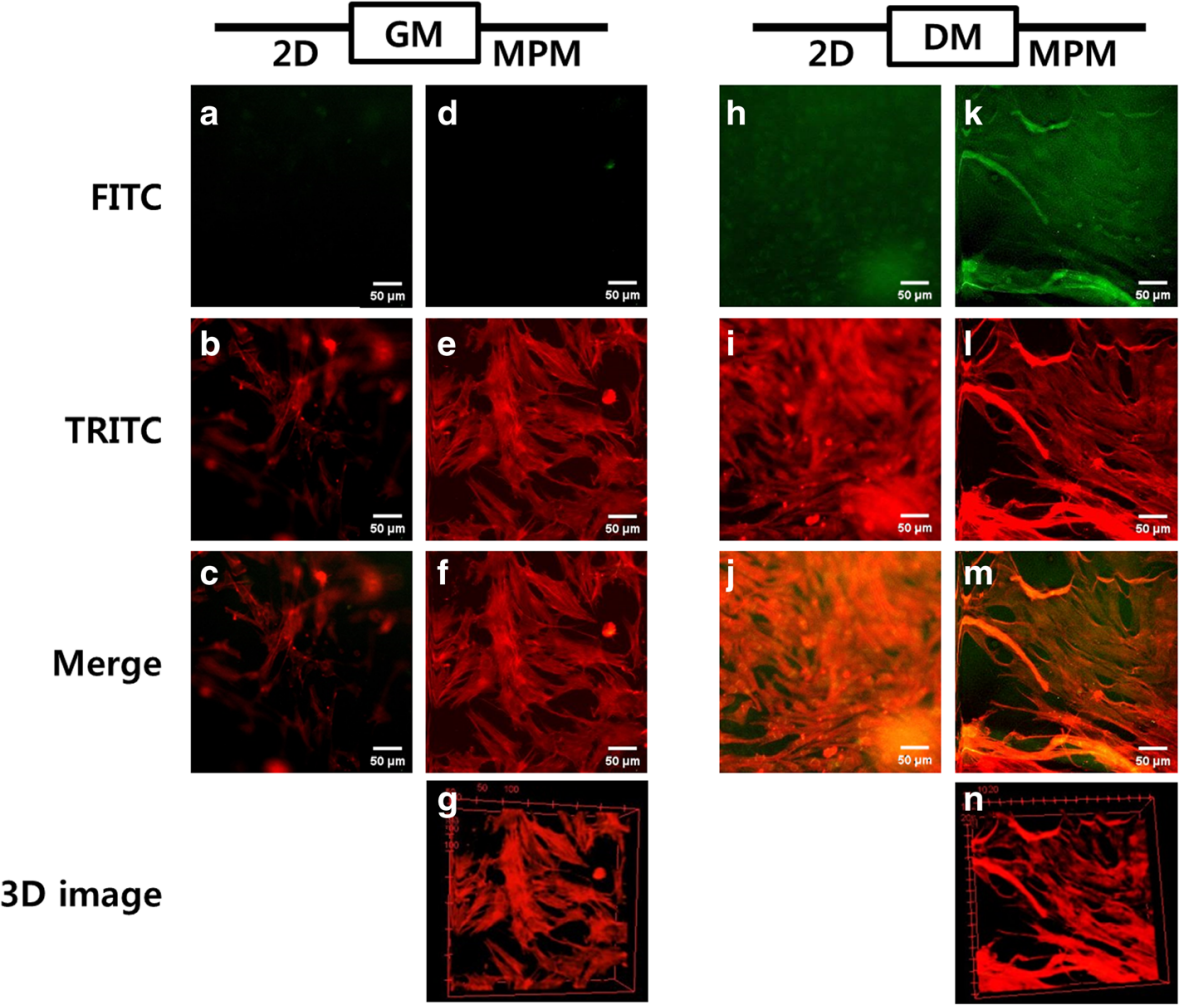
Immunofluorescence images of C2C12 cells in GHPA hydrogels. 2D fluorescence images of C2C12 myoblasts in GM (**a-c**) and DM (**h-j**) and MPM images of C2C12 myoblasts in GM (**d-g**) and DM (**k–n**)**.** The F-actins were stained with TRITC-labeled phalloidin (red) and the MHCs were stained with Alexa Fluor 488-conjugated anti-MHC monoclonal antibody (green). All images shown in this figure are representative of six independent experiments with similar results

Immunofluorescence staining analysis was conducted to examine the morphology of the C2C12 myoblasts in GM and DM, respectively. MHC, a marker for myogenic differentiation, was stained with green fluorescence and the F-actins were stained with red fluorescence [38, 39]. The cells were located at different positions and grown in 3D in the GHPA hydrogels. Therefore, not all the cells were observed at one time by conventional 2D fluorescence microscopy because the focus was settled only a flat layer. On the other hand, MPM can scan layer by layer in a short time, which leads to the construction of 3D structure images from 2D images by stacking the Z axis [39–41]. In addition, MPM used a longer wavelength than conventional 2D fluorescence microscopy, which in turn means that MPM can obtain clearer and deeper layer images than conventional 2D fluorescence microscopy. As shown in Figs. 5 (a-c), the conventional 2D fluorescence images are blurry except for the center area. In contrast, MPM images are clear and bright overall, as shown in Figs. 5 (d-f). On the other hand, MHC was not expressed in the C2C12 myoblasts when they were cultured in GM (Figs. 5 (a) and (d)). MHC was expressed only in the differentiated myotubes. Differentiated cells were observed in the 3D hydrogels (Figs. 5 (h-n)). The red fluorescence from F-actins was detected at high intensity throughout the cells, but the green fluorescence from MHC was detected at low intensity. Although the MHC was expressed in the cells, it was difficult to detect the green fluorescence of MHC by 2D fluorescence microscopy due to an unclear fluorescence signal and the image was blurry (Fig. 5(h)). On the other hand, the MPM images were not only clear, but also bright. Figures 5 (k-m) shows the differentiated C2C12 myoblasts obtained by MPM. F-actins were connected between the cells and MHC was expressed in the differentiated C2C12 myoblasts. Therefore, the MPM can detect the weak fluorescence signal of MHC more easily than conventional 2D fluorescence microscopy, and brighter and clearer images are obtained by MPM. Furthermore, 3D images were acquired by MPM to provide functional information of the 3D hydrogels as well as morphological information. Figures 5 (g) and (n) represent 3D images of the C2C12 myoblasts cultured in GHPA hydrogels. Functional information of the cells and hydrogels can be obtained from these 3D images. These results suggest that the MPM is an effective method for observing 3D scaffolds and biomedical imaging.

## Conclusions

In this study, MPM, which is based on the excitation of two-photons, was customized and the C2C12 myoblasts in GHPA hydrogels were observed. These results showed that SEM and 2D fluorescence microscopy have a high resolution, but the images provide only morphological information. On the other hand, MPM images can provide both morphological and functional information. In addition, MPM has outstanding resolution with a spatial resolution of several hundreds of nanometers. Moreover, MPM images of cells, cultured even inside the GHPA hydrogels could be obtained. Therefore, MPM is a suitable imaging system for observing and analyzing cells and 3D tissues. Overall, MPM can be applied effectively to biomedical imaging technology for tissue engineering applications.
